# Sensitivity of Photosystem II to Photoinhibition in *Chlamydomonas reinhardtii* Under Conditions of Decreasing CO_2_ Depends on a Luminal Carbonic Anhydrase

**DOI:** 10.3390/ijms27146376

**Published:** 2026-07-17

**Authors:** Vasily V. Terentyev

**Affiliations:** Institute of Basic Biological Problems, Federal Research Center “Pushchino Scientific Center for Biological Research of the Russian Academy of Sciences”, 142290 Pushchino, Russia; v.v.terentyev@gmail.com

**Keywords:** *Chlamydomonas*, carbonic anhydrase, CAH3, photosystem II, water-oxidizing complex, photoinhibition

## Abstract

Some mutants of the green alga *Chlamydomonas reinhardtii*, including the *cia3* mutant deficient in the carbonic anhydrase CAH3 in the thylakoid lumen, strongly require high CO_2_ supplementation for survival, which is usually explained by disruption at some step(s) of the carbon-concentrating mechanism. This study aimed to determine whether CAH3 contributes to photosystem II (PSII) functional stability under low CO_2_ conditions. Two wild-type *C. reinhardtii* strains and the *cia3* mutant were compared for PSII photoinhibition sensitivity, D1 protein stability, pigment content, and PSII photoprotective responses before and after short-term acclimation to low CO_2_ growth conditions. Compared with the two wild-type strains, the *cia3* mutant exhibited greater PSII sensitivity to increased light intensity after acclimation to low CO_2_. In addition, this was accompanied by more pronounced degradation of the D1 protein of PSII, indicating that the absence of CAH3 also affects the structural stability of PSII. At the same time, no differences in pigment content were observed between the strains, and a low contribution of PSII photoprotective mechanisms was detected, indicating that the growth light conditions used were non-stressful for the algal photosynthetic apparatus. The observed data indicate that CAH3 contributes to maintaining the functional and structural stability of PSII under low CO_2_, even under non-stressful light growth conditions.

## 1. Introduction

The unicellular green alga *Chlamydomonas reinhardtii* is a widely used model laboratory object for studying photosynthesis at different levels, including photosystem II (PSII). In particular, this is supported by the availability of a wide range of mutants deficient in proteins of interest. Despite the fact that *C. reinhardtii* has the ability for autotrophic growth at different external CO_2_ levels, from high (5%) to low (ambient, ~0.04%) or very low (<0.01%) concentrations [1,2], some mutants demonstrate a requirement for high CO_2_ supplementation to survive in minimal medium [1,3,4,5]. More often, these mutants have disruption(s) at various stages of the carbon-concentrating mechanism (CCM), which includes a number of HCO_3_^−^ transporters and carbonic anhydrase enzymes (CA, EC 4.2.1.1) [2]. Under low and very low CO_2_ conditions, both the transporters and CAs participate jointly in the transmembrane translocation of inorganic carbon (C_i_) inside the cell and in increasing the CO_2_ concentration in the chloroplast for the carboxylation activity of ribulose-1,5-bisphosphate carboxylase/oxygenase (RuBisCO, EC 4.1.1.39) [2], tightly packed in the pyrenoid [6]. Impairment at any CCM stage results in a significant decrease in C_i_ within cells (including the chloroplast), suppression of the Calvin–Benson–Bassham cycle due to a deficiency of substrate for RuBisCO, and subsequently a lowering of the activity of the entire photosynthetic apparatus leading to suppression of culture growth [4,7,8].

The requirement for high CO_2_ under autotrophic growth on minimal medium is also well known for the *cia3* mutant of *C. reinhardtii*, which is deficient in α-CA CAH3 [3,8] localized in the thylakoid lumen [9]. Two point mutations in the second transit peptide in the mutant block translocation of the immature CAH3 protein across the thylakoid membrane, resulting in the CAH3 absence in the lumen [9,10], despite the fact that the *Cah3* gene is expressed constitutively [5,11]. Taking into account that according to the latest data CAH3 is probably the most active CA of *C. reinhardtii* cells [12], its absence at low CO_2_ conditions obviously can be crucial for the functioning of the photosynthetic apparatus.

The involvement of CAH3 in the CCM operation inside the tubules—thylakoids penetrating the pyrenoid—was proposed initially, and this idea has been used by researchers up to date [8,13,14,15]. At the same time, incredibly high amounts of CAH3 proteins have been shown to be associated with PSII-enriched membranes [16,17,18,19,20] as well as detected in PSII-containing areas of the chloroplast [13]. Thus, the possible dual role of the CAH3 within thylakoids of *C. reinhardtii* was suggested, which has been reviewed recently [10]. In both cases the proposed enzymatic function of CAH3 is the acceleration of the dehydration reaction (HCO_3_^−^ + H^+^ → CO_2_ + H_2_O) to produce CO_2_ for RuBisCO in the pyrenoid [8,13,14,15] or to facilitate proton removal from the active site of the PSII water-oxidizing complex (WOC) by rapid conversion of H^+^ into H_2_O near proton channel outlets [17,18,19].

Regarding the activity of PSII, Villarejo et al. [16] observed that PSII in cells of the *cia3* mutant grown at high CO_2_ showed an increased level of photoinhibition compared to cells of the wild type (WT) strain CC-503 after adaptation to a very high light intensity. Addition of the well-known CA inhibitor ethoxzolamide to thylakoid membranes from WT decreased the values to those observed for preparations from *cia3* cells, indicating the possible role of the CA activity of CAH3 in the support of PSII against photoinhibition. Comparable results were obtained by Hanson et al. [21] with the use of the membrane inlet mass spectrometry approach. Inhibition of the O_2_-evolving activity of PSII in *cia3* cells compared to cells of another WT strain CC-400 (both were grown at high CO_2_), was clearly detected, while the decreasing curves of the effective quantum yield of PSII with increasing intensity of illumination were approximately the same for *cia3* and WT [21]. The most recent data obtained with PSII-enriched membranes isolated from *cia3* and WT CC-503 cells grown at high CO_2_ also indicate that continuous PSII photoinhibition is more pronounced in the case of *cia3*, which can be fully prevented by addition of recombinant CAH3 with high CA activity [20]. In addition, the CAH3 protein can be involved in the spatial organization of the WOC [22].

Under acclimation to low CO_2_ levels, *C. reinhardtii* cells rapidly induce the CCM [23], which is accompanied by a significant increase in both the expression level of genes and the accumulation of related proteins [5,11,24,25,26], including some CAs [23]. At the same time, no detectable changes were observed by researchers in the content of PSII, as well as in the effective quantum yield of PSII [2,24,27,28]. There were also no notable changes found in the content of CAH3 [26,27]. However, in cells adapted to low CO_2_ the activity of CAH3 [27], or thylakoid-localized CA activity (most probably related to CAH3) was found to be increased significantly [10]. This allows proposing an important role of CAH3 in the adaptation of PSII to such changes in CO_2_ conditions for maintaining high photosynthetic activity.

Surprisingly, to date only one work has tried to examine the question of how PSII or the complete photosynthetic apparatus in the *cia3* mutant (or other CAH3-deficient mutants) is changed after acclimation to low CO_2_ following growth at relatively optimal conditions under high CO_2_. This is in spite of the fact that CAH3 is probably the most active CA in *C. reinhardtii* cells [12]. Karlsson et al. [9] found that cells of the *cia3* mutant grown at high CO_2_ show values similar to those of the WT for photosynthetic O_2_ evolution and the effective quantum yield of PSII in response to an increase in bicarbonate concentration. While after short-term adaptation to low CO_2_ for 24 h, the *cia3* cells exhibit much lower affinity to bicarbonate under measurements of these parameters, which was explained by a possible consequence of low PSII efficiency. However, whether CAH3 deficiency directly affects PSII function and structural stability after acclimation to low CO_2_ remains unclear.

The aim of the present work was to study the influence of a similar short-term acclimation for 24 h of *C. reinhardtii* cells to a low CO_2_ level on the photosynthetic activity of PSII and its structural organization in more detail using the *cia3* mutant and two WT strains (CC-503 and CC-424) initially grown at high CO_2_. The results obtained show significantly increased sensitivity of PSII in the *cia3* mutant to photoinhibition compared to cells of both WT strains, which maintain high levels of functional and structural properties of their PSII under such CO_2_ changes. The possible role of CAH3 is discussed.

## 2. Results

### 2.1. Changes in Activity of the Water-Oxidizing Complex

The O_2_-evolving activity of PSII is one of the sensitive functional parameters of the photosynthetic apparatus responsive to the negative influence of altered growth conditions or any stress factors. Its suppression usually occurs due to changes in the stability status of the WOC on the PSII donor side. According to previously published reports, the CAH3 enzyme located in the close vicinity of the WOC supports its functional stability through high CA activity [16,17,18,19,22,29]. Thus, the absence of CAH3 near PSII in the *cia3* mutant cells can influence the WOC sensitivity to stress factors, especially in the case of C_i_ limitation, different forms of which are substrates for the enzymatic activity of CAH3.

For algal cells there is an easy approach to measure the O_2_-evolving activity of PSII separately from the rest of the electron transport chain of the thylakoid membrane by using an artificial acceptor 2,6-dichloro-1,4-benzoquinone (DCBQ) able to accept electrons directly from the PSII acceptor side, in combination with potassium ferrocyanide [30]. Such a method was applied here to study the effect of short-term acclimation of cells of two WT strains—CC-503 (WT503) and CC-424 (WT424), and the mutant *cia3* strain to low CO_2_ on the WOC function. To analyze changes in the O_2_-evolving activity of PSII in more detail, measurements were conducted in a wide range of actinic light intensities (25–3900 μmol photons m^−2^ s^−1^) to obtain so-called ‘light curves’ for each algal culture studied.

**Figure 1:**
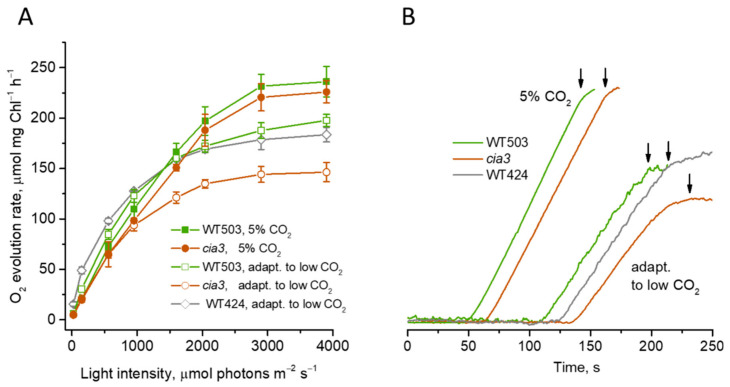
The O_2_-evolving activity of the WOC of PSII. Dependence of the O_2_ evolution rate on light intensity (**A**) and shapes of the experimental O_2_ evolution curves at the maximum applied light intensity of 3900 μmol photons m^−2^ s^−1^ (**B**) measured for two WT and the *cia3* mutant cells grown at 5% CO_2_ and adapted to low CO_2_ for 24 h. Data for WT424 grown at high CO_2_ are similar to those for WT503 and are omitted. ↓—switching off the actinic light (after ~100 s of illumination). In panel (**B**), the curves are shifted relative to the abscissa axis for better visualization.

Results indicated that cells of all studied strains initially grown at high CO_2_ were characterized by very similar shapes of the observed light curves (Figure 1A). The plateaus of the maximum activities were reached at light intensities of ~2900 μmol photons m^−2^ s^−1^ with half-maximum values at ~1000 μmol photons m^−2^ s^−1^. The O_2_-evolution rate at ~2900 μmol photons m^−2^ s^−1^ was calculated to be in the range of 220–230 μmol O_2_ mg Chl^−1^ h^−1^. These data clearly indicated that the functional stability of the WOC in PSII of the two WT and the *cia3* mutant cells was high and almost equal. This was in good agreement with the previously obtained results showing the absence of significant structural and functional differences in PSII-enriched membranes isolated from WT503 and *cia3* cells grown under optimal conditions with high CO_2_ [19].

Following short-term acclimation of the algal cultures to ambient air without supplementation of external CO_2_ (i.e., to low CO_2_) for 24 h, led to a decrease in the maximum O_2_-evolving activity of PSII, reflecting in the shapes of the observed light curves, and these characteristics were more pronounced in the case of the *cia3* mutant cells (Figure 1A). Nevertheless, the slopes on the light curves in all three strains were similarly smoother before the plateaus, which were reached at an actinic light intensity of ~2000 μmol photons m^−2^ s^−1^ with half-maximum values at ~560 μmol photons m^−2^ s^−1^, respectively. The latter was possible due to the steeper rise in the curve in the case of WT cells at light intensities up to 1000 μmol photons m^−2^ s^−1^. In both WT strains the values of the O_2_-evolution rate of PSII at ~2000 μmol photons m^−2^ s^−1^ were close and equal to ~170 μmol O_2_ mg Chl^−1^ h^−1^, which was by ~22–26% lower compared to cells from high CO_2_ conditions.

PSII in cells of the *cia3* mutant in such conditions had a maximum O_2_-evolving activity of ~145 μmol O_2_ mg Chl^−1^ h^−1^. On the one hand, this was ~15% lower compared to cells of the two WT strains. On the other hand, it was by ~35–37% lower compared to cells from high CO_2_ conditions. In addition, the WOC activity in PSII of the *cia3* mutant was characterized by a strongly reduced resistance to increasing intensities of the actinic light applied. For example, under short but extremely high light illumination (~3900 μmol photons m^−2^ s^−1^) used during the measurements, the photoinhibition of the WOC function began to be detected directly on the curve of O_2_ evolution after ~67 s with the complete suppression of the WOC activity during the next 33 s, i.e., after 100 s of total illumination (Figure 1B). Such inhibition of the WOC function was not detected in the case of both WT cells, which were also adapted to low CO_2_ for 24 h.

Therefore, the data obtained indicated the induction of a much greater functional destabilization of the WOC in PSII of the *cia3* mutant cells deficient in CAH3 in contrast to both WT under acclimation to low CO_2_ conditions even without extremely high light pre-exposure as applied in previous studies [16,20,21].

### 2.2. Pigment Composition

Previously published reports indicated the maintenance of the Chl *a*/*b* ratio in *C. reinhardtii* cells over a wide range of stress factors, including high light and CO_2_ limitation [28,31,32]. However, changes in carotenoids (Car) metabolism in response to low CO_2_ stress at moderate light intensity were identified [33], and high light stress always leads to a decrease in total Chl content with an increase in the Car/Chl ratio in *C. reinhardtii* cells [31,32,34]. In the present study, low (non-stressful) light conditions were used together with CO_2_ limitation, and pigment composition analysis can indicate whether the applied growth conditions induced any stress responses at the level of the entire photosynthetic apparatus or not.

As shown in Table 1, algal cultures of all three strains grown at 5% CO_2_ showed similar values of total Chl concentration, Chl *a*/*b*, and Car/Chl ratios, which was consistent with the similar O_2_-evolving activity of PSII detected in these algal cells (Figure 1A). In addition, the values were close to those obtained previously for other *C. reinhardtii* strains [32,35,36]. Acclimation of the two WT strains and the *cia3* mutant to low CO_2_ for 24 h, in turn, induced a stronger decrease in both the O_2_-evolving activity and high light resistance of PSII in the *cia3* mutant cells (Figure 1A). However, it did not result in changes in the concentration of total Chl, or the ratios of Chl *a*/*b* and Car/Chl compared both between strains and with the values obtained at 5% CO_2_ (Table 1). This indicated that the growth conditions used were probably not stressful for algal cultures even in the case of short-term limitation of CO_2_ and did not trigger any notable stress responses in the photosynthetic apparatus of the cells.

### 2.3. Chlorophyll Fluorescence Data

**Figure 2:**
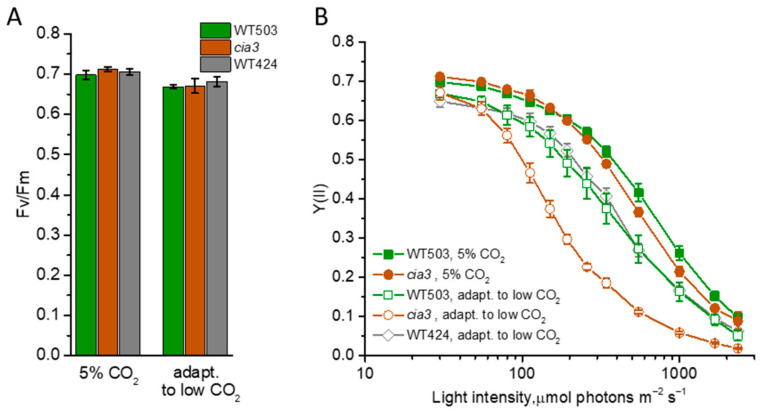
The maximum quantum yield (Fv/Fm) of PSII (**A**) and the dependence of the effective quantum yield (Y(II)) of PSII on applied light intensity (**B**), measured in two WT and the *cia3* mutant *C. reinhardtii* cells grown at 5% CO_2_ and after adaptation to low CO_2_ levels for 24 h. Data for WT424 grown at high CO_2_ are similar to those for WT503 and are omitted in panel (**B**).

The data from the measurements of variable Chl fluorescence supported the suggestion mentioned above about the absence of significant stress effects caused by short-term external CO_2_ limitation on the photosynthetic apparatus in all studied strains. Thus, the maximum quantum yield of PSII (Fv/Fm) in algal cells grown at 5% CO_2_ was similarly high in the two WT strains and the *cia3* mutant (Figure 2A). After acclimation to low CO_2_ the values of Fv/Fm also similarly decreased from ~0.70–0.71 to ~0.67. This indicated that the maximum capacity of PSII for photochemistry slightly but equally decreased in all studied strains, probably due to a reduction in the functional antenna sizes of PSII [28], without any detectable influence of the CAH3 absence in the mutant. However, during measurements of the effective quantum yield of PSII (Y(II)) reflecting the efficiency of PSII in cells adapted to light, the differences between the two WT and the *cia3* cells were observed identified (Figure 2B).

In accordance with the study of O_2_-evolving activity (Figure 1A), the analysis of the Y(II) parameter of PSII in algal cells was also conducted over a wide range of actinic light intensities (~30–2350 μmol photons m^−2^ s^−1^). The obtained results indicated that curves of the decrease in Y(II) values with the increase in light intensity (so-called here “Y(II) light curves”) were almost similar in the two WT and the *cia3* mutant cells grown at 5% CO_2_, with half-maximum values reached at light intensities of 600–700 μmol photons m^−2^ s^−1^ (Figure 2B). Acclimation of algal cultures to low CO_2_ led to changes in the Y(II) curves and, in contrast to both WT strains, the *cia3* mutant cells demonstrated more critical sensitivity to light intensity. Clear differences in the Y(II) light curves between the two WT and the *cia3* mutant cells began to be detected at light intensities above ~80–100 μmol photons m^−2^ s^−1^, with the half-maximum reached for the two WT and *cia3* cells at ~400 and ~160 μmol photons m^−2^ s^−1^, respectively. It means that in comparison with algae cultures grown at high CO_2_ these values were reached at light intensities lower by ~33% in both WT and by ~74% in the *cia3* mutant cells light intensities, i.e., the difference was more than twofold. At the same time, at a light intensity of 600–700 μmol photons m^−2^ s^−1^ where algal cells from high CO_2_ showed a half of their Y(II), the two WT and the *cia3* mutant had Y(II) values equal to ~0.25 and ~0.1 (~50% and ~20% of the 0.5 value), respectively.

The largest difference between the Y(II) light curves, obtained for the two WT and the *cia3* mutant cells adapted to low CO_2_, was observed at a light intensity of ~260 μmol photons m^−2^ s^−1^ (Figure 2B). Compared to cells grown at 5% CO_2_, the value of Y(II) at such light intensity in cells of the two WT and the *cia3* mutant was lower by ~23% and ~58%, respectively. This indicated that the photochemical activity of PSII under CO_2_ limitation was lost ~2.5-fold more strongly in the *cia3* mutant cells lacking CAH3 than in WT cells. Interestingly, such conditions are often used as a moderate light intensity in studies with *C. reinhardtii* [28,29,33].

**Figure 3:**
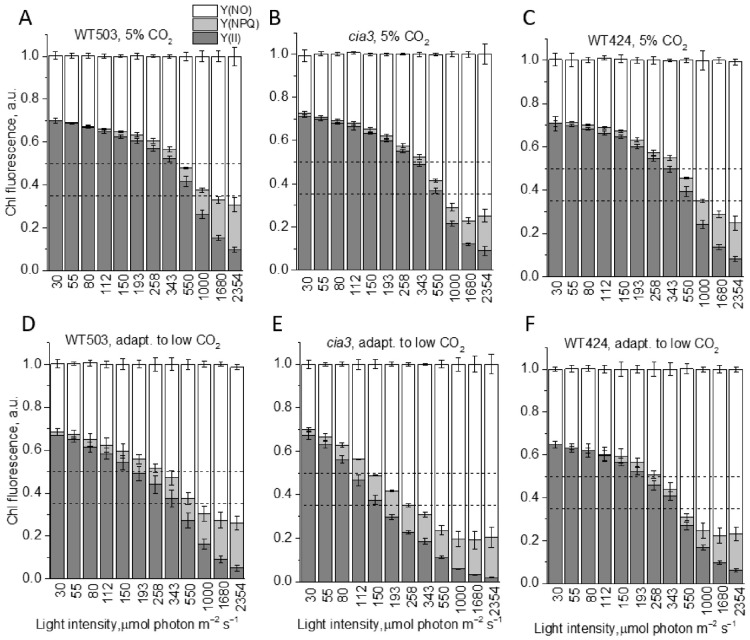
Partitioning between the effective quantum yield (Y(II)) and the quantum yields of regulated (Y(NPQ)) and non-regulated (Y(NO)) non-photochemical quenching of PSII observed at different light intensities. The data obtained for the two WT (**A**,**C**,**D**,**F**) and the *cia3* mutant (**B**,**E**) cells of *C. reinhardtii* grown at 5% CO_2_ (**A**–**C**) and adapted to low CO_2_ for 24 h (**D**–**F**). Upper dashed lines indicate a 0.5 value of the total Chl fluorescence, whereas lower dashed lines indicate a 0.5 value of Y(II).

Partitioning of absorbed light energy between photochemical quenching of PSII, represented by Y(II), and quantum yields of non-photochemical quenching (NPQ) of PSII, consisting of regulated Y(NPQ) and non-regulated Y(NO) components, at different light intensities is presented in Figure 3. A similar approach has already been used for higher plants [37], allowing comparison of the data between green algae and higher plants.

Expectedly, in all cases (the two WT and *cia3* cells grown at 5% CO_2_ and two WT and *cia3* cells adapted to low CO_2_ for 24 h) the contribution of total NPQ increased with increasing light intensity applied. In the two WT and the *cia3* mutant cells grown at 5% CO_2_ half of the absorbed light energy began to be dissipated through NPQ at close light intensities of ~350 μmol photons m^−2^ s^−1^ (Figure 3A–C). This means that before these light intensities, more than half of absorbed light energy was used for PSII photochemistry. After short-term adaptation of the algal cultures to low CO_2_, the contribution of the NPQ component became greater, and this was more pronounced in the *cia3* mutant cells. The point at which half of the total Chl fluorescence became related to NPQ was achieved at ~190 μmol photons m^−2^ s^−1^ in the case of the two WT and at ~100 μmol photons m^−2^ s^−1^ in the case of the *cia3* mutant cells (Figure 3D–F), i.e., the difference in light intensities was almost twofold. If compared to the data obtained for cells grown at 5% CO_2_, then the values of light intensities decreased by ~45% and ~71% for the two WT and the *cia3* mutant, respectively. This indicated a significant decrease in photochemical efficiency (increased photoinhibition) of PSII without CAH3 in the *cia3* mutant cells induced by acclimation to low CO_2_ conditions.

Interestingly, the main contribution to the total NPQ in the studied *C. reinhardtii* strains arose from the Y(NO) component, which is in contrast to that observed for higher plants, where Y(NPQ) was the main part of NPQ under increased light intensities [37]. In the present research the proportion of Y(NPQ) was no more than ~20–23% and its contribution to the total NPQ was obviously higher in algal cells acclimated to low CO_2_. But even in this case, at the point when the total NPQ reached half of the Chl fluorescence, the proportion of the Y(NPQ) component was only ~10–12% and ~15% for the two WT and the *cia3* mutant, respectively. This also can be explained by non-stressful low light growth conditions for PSII of algal cultures [38].

### 2.4. Western Blot Analysis of Proteins Related to PSII

**Figure 4:**
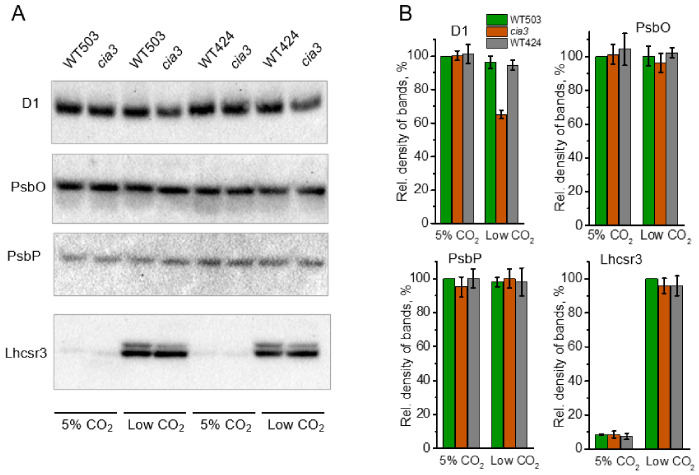
The results of Western blot analysis for the two WT and the *cia3* mutant cells grown at 5% CO_2_ and then adapted to ambient CO_2_ for 24 h with the use of the primary antibodies raised against the D1, PsbO, and PsbP proteins of PSII, as well as the Lhcsr3 protein (**A**). The relative density of protein bands (**B**) calculated in comparison to WT503 grown at 5% CO_2_ (and, in the case of Lhcsr3, in comparison to WT503 adapted to low CO_2_).

To identify if any reorganizations in proteins related to the PSII super-complex were induced by low CO_2_ in the studied algal strains, Western blot analysis was performed. The results showed that in the two WT and the *cia3* mutant cells grown at 5% CO_2_, the content of the D1 protein (one of the major proteins of the PSII reaction center), as well as the PsbO and PsbP proteins (proteins of the WOC), were very similar (Figure 4 and Supplementary Materials). This indicated a similar amount of assembled PSII complexes, which correlated well with previously observed data obtained for both whole algal cells grown at low light and 5% CO_2_ [29] and PSII-enriched membranes isolated from such cells [18,19]. In addition, this is in agreement with the functional similarity of PSII among all studied strains described above (Figure 1, Figure 2 and Figure 3).

After adaptation of algal cells to low CO_2_, the amount of the D1 protein, as well as PsbO and PsbP proteins, did not change in both WT and was close to that observed in cells grown at 5% CO_2_. This was in good agreement with previously published data obtained for different strains of *C. reinhardtii* used as WT [24,25,27,28]. However, in the case of the *cia3* mutant cells, the amount of the D1 protein has clearly decreased by more than ~35% (Figure 4 and Supplementary Materials) CC, while the amounts of PsbO and PsbP proteins of the WOC did not change and were close to those obtained for all strains grown at 5% CO_2_ and both WT cells adapted to low CO_2_. It can mean that the content of fully assembled PSII complexes in the *cia3* mutant cells adapted to low CO_2_ was lower by more than one-third compared to WT or all studied algal strains grown at 5% CO_2_. If one takes into account the balance of the PSII damage and repair cycle continuously occurring in the thylakoid membrane [39], then in the *cia3* mutant cells it can be shifted toward the D1 damage processes with retention of the WOC proteins pool in the thylakoid lumen.

Interestingly, such a decrease in the D1 content has been previously observed for different WT strains of *C. reinhardtii* after acclimation to very high light intensity [34,36,39,40], but not after adaptation to ambient CO_2_ levels [25,26,27,28]. Thus, PSII complexes in the absence of CAH3 in the *cia3* mutant cells acclimated to low CO_2_ seem to become highly sensitive even to the relatively low light intensity of the growth conditions used.

The content of the PSII-associated Lhcsr3 (Light-harvesting complex stress-related) protein, which only plays a role in the induction of regulated NPQ in *C. reinhardtii* [38], was equal in cells of all studied strains adapted to low CO_2_ (Figure 4 and Supplementary Materials). This can indicate that the contribution of regulated mechanisms of PSII protection against photoinhibition was not different in the two WT and the *cia3* mutant cells after their acclimation to low CO_2_. Algal cells grown at 5% CO_2_ contained no more than 10% of Lhcsr3, which was in good agreement with previously published data indicating the inhibition of Lhcsr3 accumulation in *C. reinhardtii* by high CO_2_ [2,26,38].

At the same time, in spite of the more than 10-fold difference in the Lhcsr3 content in cells grown at high CO_2_ and acclimated to low CO_2_, the contribution of regulated NPQ (Y(NPQ)) to the total NPQ was relatively small in all studied variants of the algal cultures (Figure 3). Even in the case of the *cia3* mutant cells acclimated to low CO_2_, in which PSII were more functionally unstable and sensitive to light (as shown by the data presented above), the regulated NPQ mechanisms were not more active. This obviously indicated that the photosynthetic apparatus in all studied *C. reinhardtii* strains in used growth conditions did not activate regulated NPQ for PSII protection, probably because the algal cells were not pre-acclimated to high light, which is usually required for NPQ induction [34,38]. Thus, PSII in the *cia3* mutant cells deficient in CAH3 after acclimation to low CO_2_ remained defenseless to light despite its increased sensitivity to it.

## 3. Discussion

The direct study of changes in the functional and structural properties of PSII induced by low CO_2_ in *C. reinhardtii* cells lacking carbonic anhydrase CAH3 of the thylakoid lumen (*cia3* or similar mutants) has not been performed previously. This is because the functional role of CAH3 is usually considered in terms of its participation in the CCM. As assumed, the CAH3 absence just decreases RuBisCO activity due to the low CO_2_ flow from the thylakoid lumen to the pyrenoid matrix and, therefore, suppresses culture growth (reviewed in [10]). Indeed, it was shown that different *cia* mutants (*cia 1–5*), including *cia3*, were not able to grow at air (low) levels of CO_2_ [3,8], which logically indicated the disruption of some CCM steps in algal cells. From this point of view, the letter designation in the mutant names meant the presence of some specific properties in inorganic carbon (*C_i_*) *a*ccumulation appearing in the phenotype [3]. However, the suggestion about the association of CAH3 with PSII on the luminal side of the thylakoid membrane arose together with the CAH3 identification, obtaining the *cah3* gene sequence, and the related protein isolation from *C. reinhardtii* cells [9,41]. The generation of the primary antibodies against CAH3 and subsequent studies with PSII-enriched membranes also supported this proposal [16,17,18,19,27]. The influence of the CAH3 absence on the PSII function was confirmed in many further works with the use of the CAH3-deficient mutant *cia3* (reviewed in [10]), as well as with a highly active recombinant CAH3 protein [12,17,20].

Data of the present work clearly indicate that even short-term acclimation of the *cia3* mutant cells to low CO_2_ induces significant changes in the functional stability of their PSII in contrast to that in cells of the two WT strains used. This appeared in a notable decrease in the resistance of PSII in *cia3* cells to increased light intensity. For example, the light curves of both the O_2_ evolution rate and Y(II) obtained for the *cia3* mutant cells showed more than twofold differences compared to that observed for both WT (Figure 1A and Figure 2B) indicating significantly higher sensitivity of PSII in *cia3* to photoinhibition. Interestingly, the decreased value of Y(II) in the case of *cia3* cells adapted to low CO_2_ was observed even at a low light intensity of ~100 μmol photons m^−2^ s^−1^ usually used for *C. reinhardtii* growth [29].

In contrast, the structural differences in PSII of the studied algal strains before and after their adaptation to low CO_2_ were not so obvious. Thus, the pigment composition in the two WT and the *cia3* mutant cells was not different from each other (Table 1) indicating not-stressful light growth conditions for the photosynthetic apparatus, as well as the absence of any perturbation in both the PSII antenna structural composition and its size. The results also showed a similar content of proteins related to the WOC (PsbO and PsbP). Only the content of the D1 protein was detected to be surprisingly lower by almost a third in the case of *cia3* mutant cells after adaptation to low CO_2_ (Figure 4). This could indicate that the balance in the PSII damage-repair cycle [39] in such cells was shifted to the ‘damage’ side, leading to disassembly of a larger portion of PSII complexes with subsequent D1 degradation but retention of the WOC proteins pool in the lumen.

One of the explanations for the observed negative effects of low CO_2_ on PSII in the *cia3* mutant can follow from the known data about specific abnormal accumulation of intracellular C_i_ by different mutants of the *cah3* gene under CO_2_ deficient conditions. In comparison with other *cia* mutants accumulating the same (*cia1*, *cia2*, *cia4*) [3] or ~6 times less (*cia5*) intracellular C_i_ under low CO_2_ [4], the *cah3* gene mutants were able to accumulate it at levels more than 4–13 times higher than those in WT cells, i.e., up to 7.6–13.2 mM [3,7,42] (also see review [10]). As shown previously, high concentrations of C_i_ can induce more pronounced photoinactivation of PSII in cells of green algae [43] and cyanobacteria [44], as well as provide a negative effect on the activity of PSII in preparations isolated from higher plants [45,46]. Inhibition of the photosynthetic activity of PSII in membrane preparations isolated from the *cia3* mutant by increased concentrations of added bicarbonate was also presented in previously published works [17,18] in spite of the fact that the algal cultures were grown at high CO_2_ conditions.

Additionally, taking into account that the pH of the chloroplast stroma in light is equal to 7.8–8.0, C_i_ should be present there mainly in the bicarbonate form [18]. In a recent study, Brinkert et al. [47] showed a requirement for the photoinduced dissociation of a bicarbonate ion from the binding site near the non-heme Fe on the PSII acceptor (stromal) side to shift the midpoint potential of Q_A_/Q_A_^−●^ to more positive values leading to an increase in the energy gap between the P^+●^ Pheo^−●^ and P^+●^ Q_A_^−●^ states (where P is the primary electron donor, Pheo is pheophytin, and Q_A_ is the primary quinone acceptor). This, in turn, makes the back reaction to P^+●^ Pheo^−●^ less favorable and reduces the possibility of chlorophyll triplet-mediated singlet oxygen (^1^O_2_) formation [47]. The very high concentration of bicarbonate in the stroma of the chloroplast can obstruct the bicarbonate ion dissociation from the binding site near the non-heme Fe, which should lead to an increase in the ^1^O_2_ production. As is known, one of the first targets for reactive oxygen species in PSII is the D1 protein resulting in its oxidative damage and rapid degradation through the activity of various proteases (see review [48]). The decrease in D1 content was detected by Western blot analysis in the *cia3* mutant cells after acclimation to low CO_2_ in contrast to both WT (Figure 4).

Thus, the inhibition of phototrophic growth of the *cia3* mutant cells at low CO_2_ with a high probability most likely originates not from the intracellular C_i_ deficiency and reduced RuBisCO carboxylase activity, as is now assumed, but from its significant excess, which can provide strong suppression of PSII function together with the induction of D1 degradation. As a result, this leads to the increased sensitivity of PSII to photoinhibition clearly shown in this study. Additional factors may arise from the presence of some conformational changes in PSII of the *cia3* mutant observed earlier as a result of the CAH3 subunit absence near the WOC [22].

Data about the Lhcsr3 protein content in the studied cells, first of all, indicated that acclimation to low CO_2_ was indeed completely achieved in all studied strains, as the synthesis of the Lhcsr3 protein was clearly increased (Figure 4). At the same time, there was no significant difference in the total accumulation of Lhcsr3 protein between the two WT and the *cia3* mutant cells, which, at first glance, was in contradiction to the suggestion about the presence of high intracellular C_i_ in the *cia3* mutant cells mentioned above. According to the recent reports [49,50], the regulation of the *lhcsr3* gene expression is a complicated process including several relatively distant ways of signaling. It is assumed, that it mainly occurs through the nucleus-localized CIA5 regulator, which is suppressed by high C_i_ [49,50]. However, the suggestion about the involvement of reactive oxygen species generated in the photosynthetic electron transport chain of the chloroplast in that regulation was also discussed [49]. Moreover, this can explain the experimental observation of Lhcsr3 protein accumulation at high light even under high CO_2_ conditions [38]. Therefore, the Lhcsr3 presence in the *cia3* mutant cells acclimated to low CO_2_ (probably containing high intracellular C_i_) can also be a result of the increased ^1^O_2_ production in PSII, as it was proposed above as a reason for the higher degree of D1 degradation (Figure 4). Of course, this proposal needs further detailed study.

The involvement of Lhcsr3 in regulated NPQ induction in *C. reinhardtii* requires conversion of the protein into a phosphorylated form for its correct localization within the antenna complex of PSII, which usually occurs under the action of stress factors such as high light [51]. Figure 4A shows that in the two WT and the *cia3* mutant cells acclimated to low CO_2_ the phosphorylated form of Lhcsr3 (upper band) is much less abundant compared to the unphosphorylated form (lower major band), which is in good agreement with the very low contribution of Y(NPQ) into total NPQ in all studied variants of algal cells (Figure 3). Therefore, in spite of the fact that PSII in the *cia3* mutant cells are more sensitive to the action of light, the standard mechanisms of PSII photoprotection are not activated, probably, due to the low light intensity applied for the culture growth.

Thus, the present study demonstrated much lower functional stability of PSII in the CAH3 deficient mutant *C. reinhardtii cia3* induced by even short-term acclimation to low CO_2_ growth conditions in contrast to the two WT strains. It appears as a decreased resistance of PSII activity to increasing light intensities (sensitivity to photoinhibition), accompanied by greater degradation of the D1 protein. Together, these results are in agreement with the suggestion about the significant involvement of carbonic anhydrase CAH3 in supporting the high functional stability of PSII in *C. reinhardtii* cells.

## 4. Materials and Methods

### 4.1. Algal Strains and Growth Conditions

The cell wall-less mutants (*cw*) of *C. reinhardtii* were used in the study. According to previously published works strains CC-503 (*cw92*) and CC-424 (*cw15*) were taken as two WT (WT503 and WT424) and *cia3* was used as the mutant deficient in CAH3 in the thylakoid lumen [3,9,16,17,18,19,27,29]. Algal cultures were grown photoautotrophically in parallel in TAP liquid medium at 25 °C under aeration by air enriched with 5% CO_2_ until the mid-exponential phase was reached. For acclimation of algae to ambient CO_2_ levels, the enrichment of the air with CO_2_ was stopped. The medium for CC-424 was supplemented with 100 mg/L arginine through 0.22 μm syringe filter [52]. Continuous illumination was provided by LED lamps with cool light (6200 K) at 90–100 μmol photons m^−2^ s^−1^.

### 4.2. Pigment Content Determination

Concentrations of total Chl and Car, as well as Chl *a* and Chl *b* content were determined spectroscopically according to [53] after pigments extraction in 80% acetone.

### 4.3. Oxygen Evolution Measurements

The rate of photosynthetic oxygen evolution by PSII in living algal cells was measured at 25 °C with a Clarke-type electrode in a 1 mL cell (Hansatech Instruments Ltd., Norfolk, UK), using 0.2 mM 2,6-dichloro-1,4-benzoquinone (DCBQ) and 1 mM potassium ferricyanide (FeCy) as artificial electron acceptors [30] and illumination with red light (≥600 nm) at different light intensities. Before measurements, cell suspensions were diluted with growth medium to a Chl concentration of 20 µg mL^−1^.

### 4.4. Chlorophyll Fluorescence Measurements

PSII photosynthetic parameters based on Chl fluorescence were measured with a MULTI-COLOR PAM fluorometer (Waltz, Effeltrich, Germany). The maximum quantum yield of PSII was calculated as Fv/Fm, where Fv = Fm − Fo, Fo is the initial fluorescence and Fm is the maximum fluorescence value obtained with saturating 200 ms flash (λ = 625 nm, ~12000 μmol photons m^−2^ s^−1^) in 20–30 min dark-adapted cells. The effective quantum yield (Y(II)) of PSII and quantum yields of regulated (Y(NPQ)) and non-regulated (Y(NO)) non-photochemical quenching of PSII were calculated as: Y(II) = (Fm’ − Fs)/Fm’, Y(NPQ) = Fs/Fm’ − Fs/Fm, and Y(NO) = Fs/Fm, where Fmʹ is the flash-induced maximum fluorescence and Fs is the stationary fluorescence level in light (λ = 625 nm) adapted algal cells. Measurements were performed in 10 × 10 mm quartz cuvette (Hellma Analytics, Müllheim, Germany) with average stirring at room temperature in the same medium and at Chl concentration as it was used in the measurements of oxygen evolution.

### 4.5. SDS-PAGE and Western Blot Analysis

Cell suspension corresponding to 15 µg Chl was collected for each sample, after that the cells were precipitated by centrifugation at 5000 rpm for 5 min. Total protein extracts of the cells were prepared in loading buffer containing 50 mM Tris-HCl (pH 6.8), 3% SDS, 10% sucrose, 5% β-mercaptoethanol, and 0.005% bromophenol blue with final volume of 100 µL with subsequent heating at 95 °C for 3–4 min. Samples were centrifuged again at 12,000 rpm for 5 min and supernatants were loaded to the 12.5% gel at equal Chl concentration of 1.5 µg per track. SDS-PAGE and Western blot were performed as described previously [18,19] with the use of rabbit primary antibodies raised against D1 (PsbA), PsbO, and PsbP proteins of PSII, as well as against Lhcsr3 proteins, produced by Agrisera (Vännäs, Sweden) (AS111786, AS06 142–33, AS06 142–23, and AS14 2766, respectively).

### 4.6. Statistical Analysis

All measurements were performed with at least three biological replicates and at least three technical replicates. Statistical analysis was performed using the standard algorithms of OriginPro (2016) (OriginLab, Northampton, MA, USA). The data are presented as the mean ± SD. Differences with *p*-values < 0.05 were considered statistically significant.

## Figures and Tables

**Table 1:** Pigment composition of algal cells grown at 5% CO_2_ and adapted to low CO_2_ for 24 h. Letters without apostrophes, with one apostrophe, or with two apostrophes indicate statistically significant differences between values within each parameter, *p* < 0.05.

	Total Chl, μg mL^−1^		Chl *a*/*b* Ratio		Car/Chl Ratio	
	High CO_2_	Low CO_2_	High CO_2_	Low CO_2_	High CO_2_	Low CO_2_
WT503	15.1 ± 1.8 ^a^	14.0 ± 1.5 ^a^	2.78 ± 0.09 ^a’^	2.90 ± 0.11 ^a’^	0.28 ± 0.003 ^a”^	0.28 ± 0.003 ^a”^
*cia3*	15.3 ± 1.9 ^a^	16.5 ± 0.5 ^a^	2.84 ± 0.07 ^a’^	2.75 ± 0.09 ^a’^	0.28 ± 0.002 ^a”^	0.27 ± 0.005 ^a”^
WT424	14.1 ± 1.1 ^a^	15.0 ± 1.0 ^a^	2.80 ± 0.07 ^a’^	2.91 ± 0.04 ^a’^	0.28 ± 0.004 ^a”^	0.27 ± 0.021 ^a”^

## Data Availability

The original contributions presented in this study are included in the article/Supplementary Materials. Further inquiries can be directed to the corresponding author.

## Supplementary Materials

The following supporting information can be downloaded at: https://www.mdpi.com/article/10.3390/ijms27146376/s1.
