# Supplementary material for: Sensitivity of Photosystem II to Photoinhibition in Chlamydomonas reinhardtii Under Conditions of Decreasing CO2 Depends on a Luminal Carbonic Anhydrase
Source: Int J Mol Sci. 2026 Jul 17;27(14):6376. doi: 10.3390/ijms27146376 (PMC13410348; doi:10.3390/ijms27146376)
Supplement: Supplementary file 1 [file ijms-27-06376-s001.zip › ijms-4374834-supplementary.pdf]

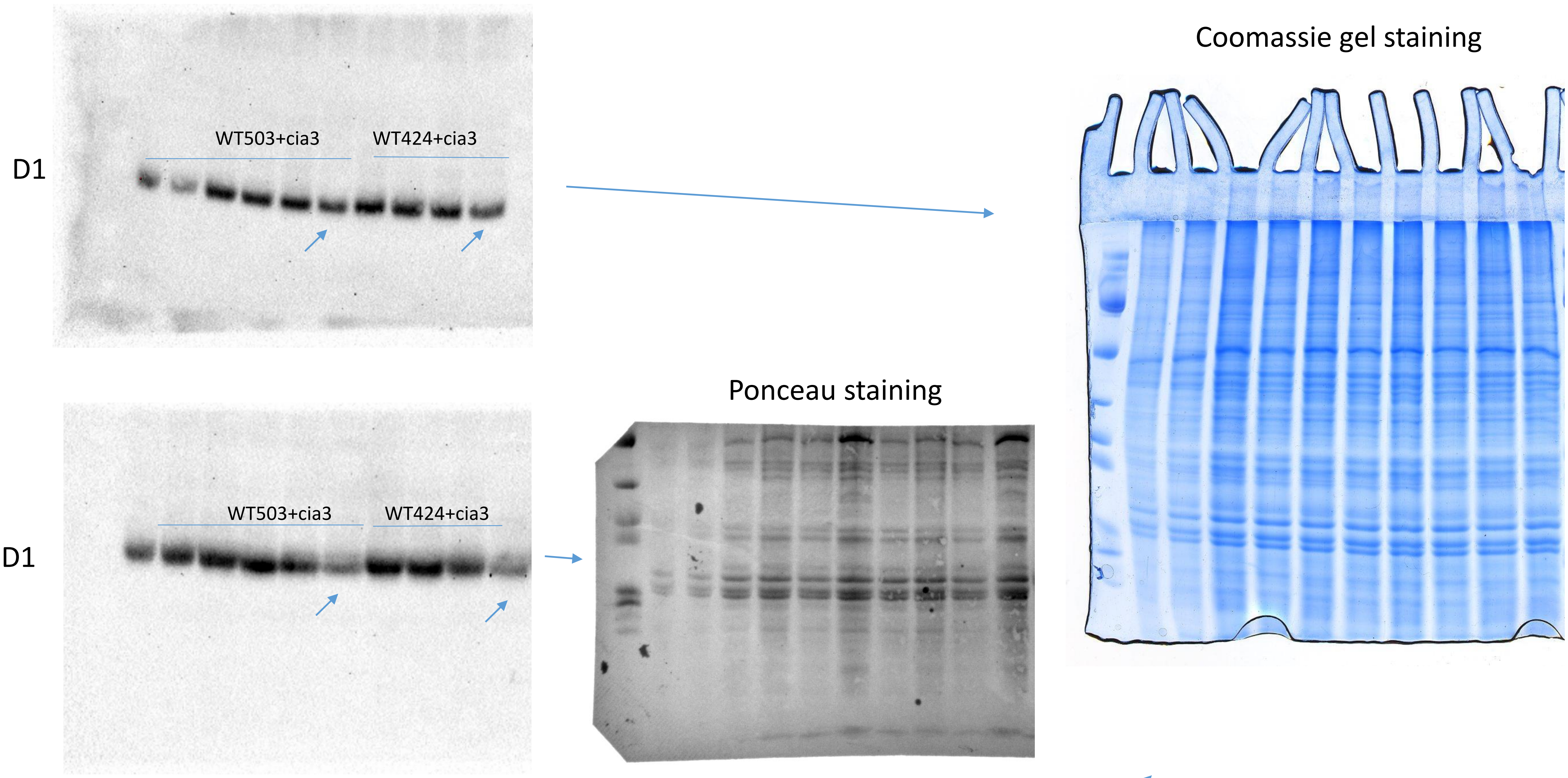

- *cia3* under low CO_2 conditions

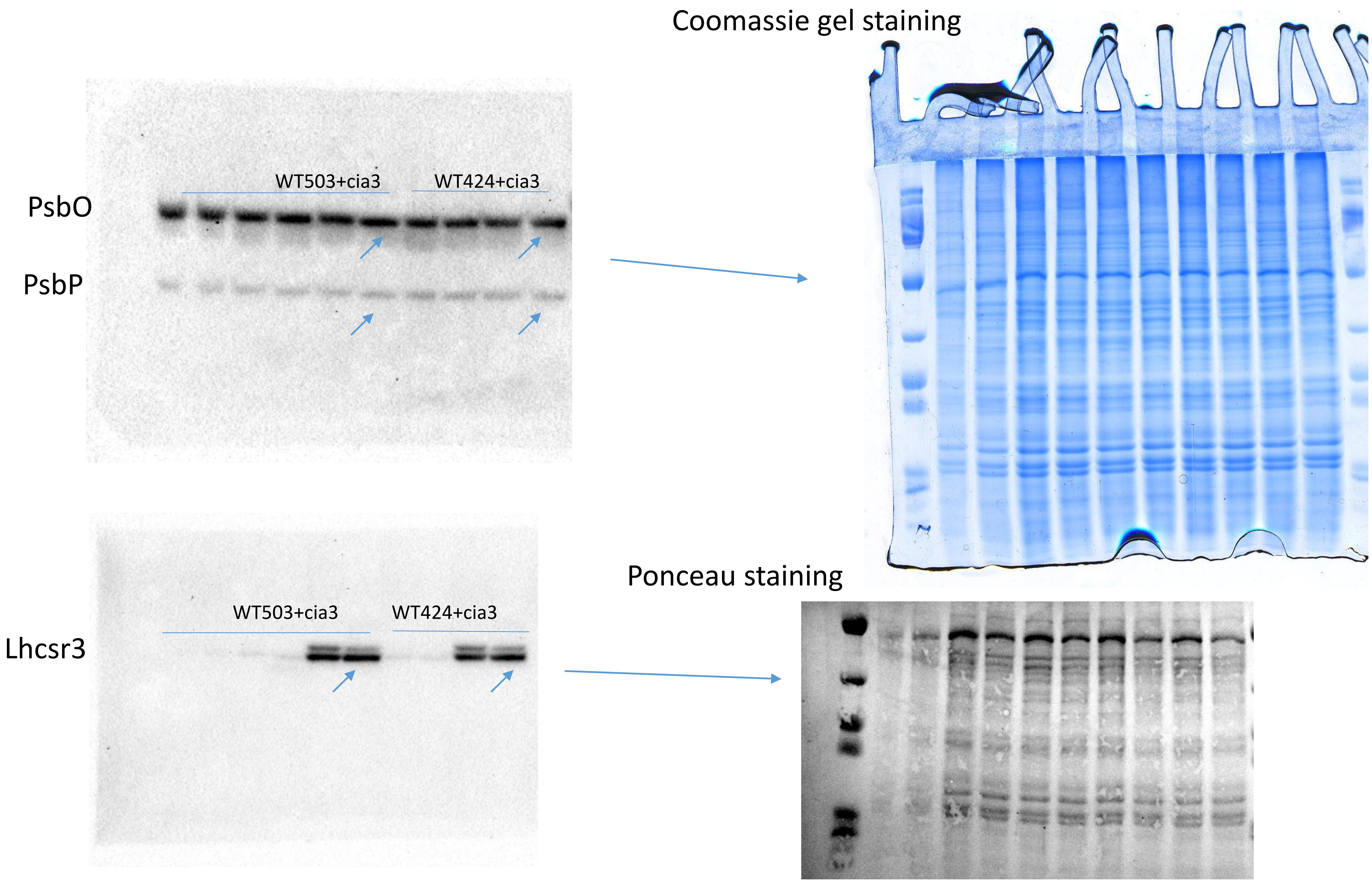
Coomassie gel staining
WT503+cia3
WT424+cia3
PsbO
PsbP
Ponceau staining
WT503+cia3
WT424+cia3
Lhcsr3
